# Nanocomposite Membranes for Liquid and Gas Separations from the Perspective of Nanostructure Dimensions

**DOI:** 10.3390/membranes10100297

**Published:** 2020-10-21

**Authors:** Pei Sean Goh, Kar Chun Wong, Ahmad Fauzi Ismail

**Affiliations:** Advanced Membrane Technology Research Centre (AMTEC), School of Chemical and Energy Engineering, Faculty of Engineering, Universiti Teknologi Malaysia, Johor Bahru 81310, Malaysia; amtec@utm.my (K.C.W.); afauzi@utm.my (A.F.I.)

**Keywords:** nanocomposite membranes, inorganic nanomaterials, nanomaterials, liquid separation, gas separation

## Abstract

One of the critical aspects in the design of nanocomposite membrane is the selection of a well-matched pair of nanomaterials and a polymer matrix that suits their intended application. By making use of the fascinating flexibility of nanoscale materials, the functionalities of the resultant nanocomposite membranes can be tailored. The unique features demonstrated by nanomaterials are closely related to their dimensions, hence a greater attention is deserved for this critical aspect. Recognizing the impressive research efforts devoted to fine-tuning the nanocomposite membranes for a broad range of applications including gas and liquid separation, this review intends to discuss the selection criteria of nanostructured materials from the perspective of their dimensions for the production of high-performing nanocomposite membranes. Based on their dimension classifications, an overview of the characteristics of nanomaterials used for the development of nanocomposite membranes is presented. The advantages and roles of these nanomaterials in advancing the performance of the resultant nanocomposite membranes for gas and liquid separation are reviewed. By highlighting the importance of dimensions of nanomaterials that account for their intriguing structural and physical properties, the potential of these nanomaterials in the development of nanocomposite membranes can be fully harnessed.

## 1. Introduction

Membrane-based separation has earned its place in a wide spectrum of commercial applications. Over the last few decades, progressive growth has been witnessed in the application of membrane technology in wastewater treatment, desalination, gas separation and energy generation [1,2,3]. With its commercial attractiveness based on advantages such as high energy efficiency, low carbon footprint and operational simplicity, attaining a reliable and sustainable separation process based on membrane technology is one of the greatest areas of interest in this field [4,5]. The membrane is known as the heart of the entire separation process. The separation performance and efficiencies are closely related to the intrinsic properties of membranes. A highly selective membrane material ensures high purity; a highly permeable membrane material allows sufficiently high productivity for large-scale application; a fouling resistant membrane material extends the shelf lifespan of the liquid separation membrane module which leads to cost saving. Although polymeric membranes are currently dominating the membrane market, there are intensive efforts to develop high-performance membranes with multifunctional physicochemical properties [6,7].

Nanotechnology has been dynamically adapted to a broad range of modern applications, including membrane development. The emergence of nanoscience is also concerned with the production of new or enhanced materials. Nanomaterials are the subject of intense research and are known to be highly versatile materials, in which their physical and chemical properties can be flexibly tailored [8]. At the atomic level, this can be accomplished by tuning the elemental composition, atomic/molecular arrangement and nanomaterial dimension via a bottom-up approach [9]. At a macroscopic level, post-synthesis modifications can be performed on the device or system to create new functionalities and capabilities. The interdisciplinary research of material chemistry and engineering applications hold the key to heightened material performance. Tapping these new opportunities, innovations have been constantly made to resolve the pertinent issues of membrane processes, including addressing the trade-off between selectivity and productivity as well as membrane fouling, membrane aging and plasticization [10]. One such innovation which has created new horizons in membrane-based separation processes is the development of nanocomposite membranes—an emerging generation of membrane which amalgamates polymers and inorganic materials as one entity [11]. The application of nanocomposite membranes has been extended in various separation processes and has made great strides in performance enhancement. The interplay between the two entities uniquely combine the benefits of each component and diminish their inherent limitations [12].

In the so-called nanocomposite membranes, the nanomaterials can be integrated with polymer matrix in several ways, including (i) the most common direct blending of nanomaterials with polymers to form mixed matrix membranes (MMMs) [13] and (ii) the post-incorporation of nanomaterials onto the preformed membrane surface through surface assembly, coating or grafting [14]. For thin-film composite membranes used for gas separation, nanofiltration (NF), reverse osmosis (RO) and forward osmosis (FO), the nanocomposite membranes can be prepared in more flexible manners, i.e., by incorporating the nanofillers into the substrate and/or the selective layer and by surface architecture [15]. Regardless of the structures and configurations, nanocomposite membranes have been hailed as one of the game changers in membrane technology. One of the most critical aspects in the development of nanocomposite membrane is a proper selection of both nanomaterials (dispersed phase) and polymer (continuous phase). Defining a set of questions related to material selection, such as what is the intended use and what the specific properties of the membrane are needed for that use can provide fruitful guidelines to visualize the outcome of the function-led membranes. Understanding the properties of materials is particularly crucial, not solely from a fundamental point of view, but also because knowledge in this aspect forms a strong basis for the development of nanocomposite membranes and their resultant performance. It is also of paramount interest for tailoring the membrane properties. The advent of characterization techniques provides adequate information about a newly discovered nanomaterial, hence helping to establish function-structure relationship. 

Nanomaterials can be characterized by multiple parameters including the dimension, surface chemistry and crystal structures. A broad classification based on their geometry and dimension allow all nanomaterials to be classified into four groups i.e., zero-dimension (0D), one-dimensional (1D), 2-dimensional (2D) and three-dimensional (3D) [16,17,18]. Accordingly, 0D nanomaterials are mostly spherical or quasi-spherical, dots and clustered nanoparticles with all the dimension confined within nanoscale below 100 nm; 1D nanomaterials are commonly featured as nanotubes, nanorods and nanowires where one of their dimensions is beyond 100 nm. The typical examples of 2D nanomaterials are sheet-like graphene and hexagonal boron nitride with only one dimension falls within single or few atomic thicknesses. In 3D nanomaterial such as polycrystal and microporous framework assemblies, all dimensions of the structure are in the microscale range. In the context of nanocomposite membranes, the surface chemistry of the nanomaterials used as a nanofiller or surface modifier of membranes such as their surface charges, hydrophilicity, functional groups are critical aspects that affects the physicochemical properties of the resultant membranes [19,20]. On the other hand, the dimension of nanostructures imparts more significant influence on the nanomaterial distribution patterns, the transport behavior across the nanofiller, as well as the interactions and the accessibility of the nanostructures to the surrounding matrix or species. Understanding of the structural properties and uniqueness is an important tool for the development of a nanocomposite membrane that serves that right purpose. While the surface chemistry aspect, particularly that related to surface functionalization or modification of nanomaterials, has been comprehensively covered in considerable review articles, the important roles of nanostructure dimension has not been given equal emphasis.

Considering the relevance of nanocomposite membrane in heightening membrane separation performances, a rich literature on the preparation and performance evaluations of nanocomposite membranes for various membrane-based liquid and gas separation processes already exists. Most reviews put more emphasize on the progressive advances in the development of nanocomposite membranes. The preparation and applications of nanomaterials with different dimensions have been comprehensively reviewed [21,22,23]. The potentials of 2D materials in enhancing the membrane separation performance are among the most discussed topics [24,25,26,27,28,29]. With the existing contributions, this review does not intend to provide an exhaustive review on the preparation and performance evaluation of nanocomposite membrane-containing nanomaterials of various dimensions, but it is aimed to outline the importance of selecting a nanostructure with an appropriate dimension that caters for its application. A brief overview of nanostructures commonly used for water and gas separation nanocomposite membranes is first presented based on their dimensional uniqueness. The correlations between nanostructure dimensions and the intended application of the nanocomposite is discussed based on some recent exemplary works. The synergistic effects of hybrids made from two or more dimensionally different nanostructures as well as the orientation of 1D and 2D anisotropic nanomaterials in the nanocomposite membranes are also discussed. Finally, some research directions that are essential for the further advancement of nanocomposite membranes with emphasis on the dimensional advantages are highlighted.

## 2. Dimension Plays Its Role: An Overview

**Figure 1:**
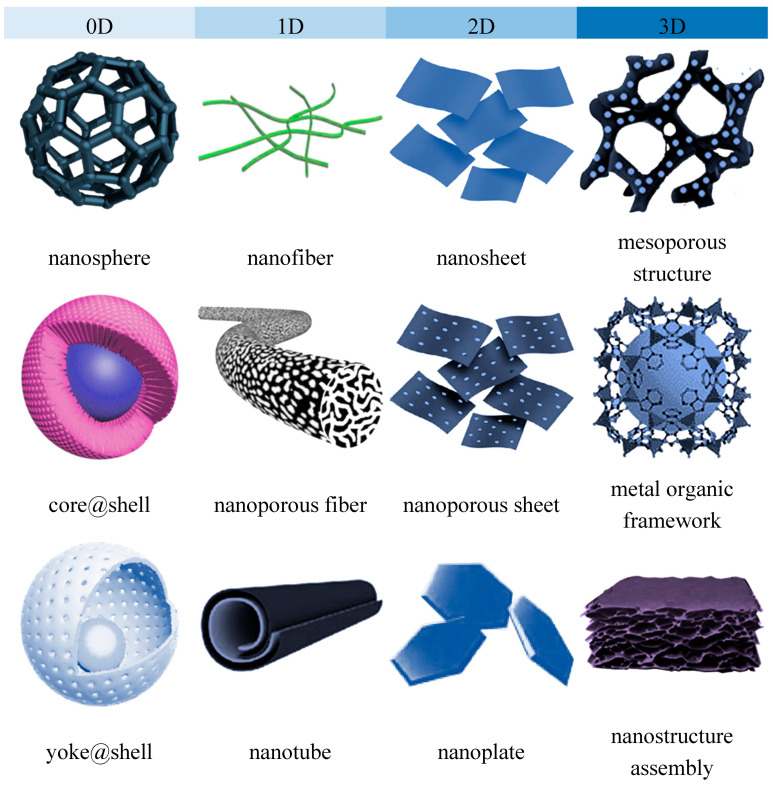
Representative illustrations of dimensionally different nanomaterials applied for gas separation and liquid separation nanocomposite membrane.

**Figure 2:**
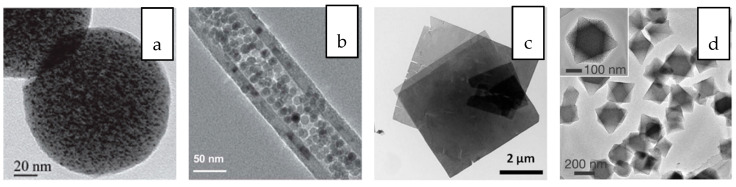
Transmission electron microscope (TEM) images of (**a**) 0D platinum loaded N-doped mesoporous carbon nanosphere [34], (**b**) 1D iron oxide nanoparticle filled carbon nanotube [35], (**c**) 2D metal organic framework nanosheet [36] and (**d**) 3D platinum loaded in between a MIL-101(Cr) core and MIL-101(Fe) shell [37].

Although the chemical composition remains unchanged, the structural and chemical parameters are significantly influenced by their dimensions, i.e., geometries and structures [30]. The most classical example is carbon element which shows unique allotropy- the existence of a range of distinctive molecular structures from the same element [31]. The allotropes exist in multidimensions, including 0D fullerence, 1D carbon nanotube (CNT), 2D graphene and 3D graphite. These carbon allotropes, especially the low-dimensional CNT and graphene have been extensively applied for the development of nanocomposite membrane owing to their outstanding novel features [32]. Due to the variation in their structural geometries as well as the chemical bonds and interactions within the structure, these allotropes are characterized by drastically different chemical properties. Nanostructured titanium dioxide or titania also can also be synthesized in several dimensions, the 0D spherical nanoparticle, 1D titania nanotubes and 2D titania nanofilm as well as the more complex hierarchical 3D titania with interconnected networks [33]. Compared to the commercially used 0D spherical nanoparticles, titania nanostructures with higher dimensions are known to feature high accessible surface areas. Figure 1 and Figure 2 present the structural illustrations and transmission electron microscope (TEM) images, respectively, of some representative dimensionally different nanomaterials applied for gas separation and liquid separation nanocomposite membrane.

The performance of nanocomposite membranes in liquid- and gas-separation applications depends critically upon the roles of the incorporated nanofillers. Together with many other interrelated factors, the geometry of the nanomaterial is important to dictate the ultimate performance of a nanocomposite membrane. The discrepancy in the performance of nanocomposite membranes incorporated with structurally different but with identical chemical composition highlights the importance of material architecture. Due to this reason, the structural features should be carefully identified. These features, such as pore size and distribution, interconnectivity of pores, and interlayer spacing, play critical roles in maximizing the desired performances. The different dimensions of these nanomaterials open rooms to develop intriguing functionalities. The tunable anisotropy in transport properties and sieving properties are two aspects closely related to membrane-separation mechanisms. The understanding on the role of nanomaterials associated to their dimension and geometry should be a subject of high interest from the material science point of view. With the advances in bottom-up and top-down synthesis, nanostructures can be feasibly synthesized in various dimensions without altering their chemical composition. In this section, the classification of the nanostructures is primarily based on their most commonly known and synthesized dimensions for nanocomposite membrane applications.

### 2.1. Zero-Dimensional Nanomaterials

Zero-dimensional (0D) nanostructures are known as the forerunner of nanostructured materials. A broad range of materials can be feasibly synthesized in spherical geometry, including carbon-based materials, metals, metal oxides, semiconductors and polymers. Zero-dimensional nanostructures are the simplest building block for the design and construction of other low-dimensional materials or complex 3D nanostructures [38]. Due to the inherent structural properties of 0D nanostructures in spherical or quasi-spherical geometry, they are characterized by ultra-small size, high surface-to-volume ratio, high active edge sites per unit mass. Owing to the diameter of less than 100 nm, 0D nanoparticles exhibit edge and quantum confinement effects that are closely related to the electrical, optical, biological, mechanical behavior and other intrinsic properties of the material [39]. Most size and dimension of all 0D nanoparticles have notable effects on the physicochemical and biological properties as these factors alters the specific surface area and thermodynamic characteristics of the nanoparticles.

Nobel metallic nanoparticles are ultrafine particles with distinguishable features from the macroscopic bulk metal. Silver nanoparticles (AgNPs) are one of the most reported antibacterial nanomaterials for the preparation of biofouling-resistant nanocomposite membranes. AgNPs can be successfully utilized as antimicrobial agents, based on their multiple bacteria-killing routes [40]. Metal oxide nanoparticles such as TiO_2_, MgO, ZnO and Al_2_O_3_ are the most common type of nanostructures used for nanocomposite membranes for both gas and liquid separation. Most of these metal oxide nanoparticles are characterized by similar physicochemical properties including large surface area, high mechanical and chemical stability. Some metal oxide nanoparticles, such as CuO [41] and ZnO [42] also exhibit strong antimicrobial activities that are comparable to that of AgNPs. The solid core of these spherical nanoparticles is impermeable so the transport of molecules across the structure has not been reported [43,44]. The surface hydrophilicity of these nanoparticles is one of the main reasons that makes them favorably used in the preparation of liquid-separation membranes. The hydrophilicity rendered by these nanoparticles improves the water flux and fouling resistant of the resultant nanocomposite membranes. On the other hand, the affinity and interaction between metal or metal oxide surface and some gas species such as CO_2_ motivates their potential for nanocomposite membranes [45,46,47]. 

Quantum dots are luminescent semiconductor crystals with diameter ranges from 1–10 nm [48]. Carbon-based quantum dots are typically 0D spherical nanoparticles with amorphous to nanocrystalline cores [49]. Compared to the semiconductor counterpart, carbon-based quantum dots exhibit lower toxicity, higher biocompatibility, better solubility and rich chemistry. Graphene and graphene oxide (GO) quantum dots are emerging 0D nanostructures that found vast applications on account of their electronic and optical properties resulted from their large edge effects and quantum confinement [50]. They are defined as graphene or GO sheets in single or several layers with lateral dimensions less than 100 nm [51]. They inherit the unique properties of monolayer sp^2^ carbon atoms structure and quantum effects of 0D nanostructure. Besides exhibiting attractive composition-related properties that fit membrane application well, the nanoscale size and homogeneity of carbon-based quantum dots also ensure their good dispersibility in polar solvents and polymer matrix [52,53,54]. 

### 2.2. One-Dimensional Nanomaterials

One-dimensional (1D) nanomaterials are characterized by hollowed tubular or solid fiber structures. All 1D nanomaterials demonstrate significantly high length to diameter aspect ratio in tandem with their tubular structure. Carbon-based 1D nanomaterials, with carbon nanotubes (CNTs) as the most classical representative of this class, are the most explored 1D nanomaterials. Structurally, CNTs can be described as hollow cylindrical tubes made up from nanometer-scale rolled up sheets of graphene that are held together by Van der Waal interactions. Carbon nanowires and nanofibers which are synthesized in the form of solid rather than hollow tubes also gained similar prominence after CNT. Naturally found inorganic analogs of carbon-based tubular nanostructures are imogolite nanotubes (INTs) and halloysite nanotubes (HNTs) [55]. INTs are aluminosilicate clay minerals known as the clay counterpart of CNTs due to their similarity in terms of aspect ratio and rigidity. The single-walled imogolite nanotube consists of two-layer where the external aluminium octahedral layer is connected to the internal silica tetrahedral layer via the covalent bonding between the mutual oxygen atoms. INTs have external diameter of about 2nn and tube length that can be extended in the range of micrometer. 

Also known as naturally occurring alumina-silicate clay, silicon oxygen tetrahedron and alumina oxygen octahedrons in HNT form a kaolinite-like sheet that rolls up into a tubular structure. Boron nitride nanotube (BNNT) is also a close structural analog of CNTs. The external diameter of BNNTs varies from 4 to 300 nm and the tube length is usually in the range of 5–10 µm [56]. The tubular structure of BNNTs is similar to that of CNT, with alternating boron and nitrogen atoms, instead of carbon, arranged in a hexagonal lattice [57]. Depending on the synthesis condition, a single-walled, double-walled or multi-walled BNNT can be obtained. Because of the structural similarity, BNNTs and CNTs shared many common features including mechanical properties and thermal conductivity [58]. Fascinated by the structural properties of these 1D nanostructures, various materials particularly metal oxide, have been synthesized in tubular nanostructures. Titania nanotubes (TNT), alumina nanotubes, zinc nanotubes, just to name a few, can be prepared through well-established templating methods [59]. 

For the development of nanocomposite membranes, in contrast to 0D nanostructures that are known to be impermeable to water or gas molecule transports, hollowed 1D nanostructures are mainly used to facilitate fast transport, the mechanisms by which a fluidic molecule is transported in the confined tubular structure responsible to the overall permeability of the resultant nanocomposite membranes [60]. The transport and thermodynamic properties of molecules across tubular structure of 1D nanomaterial is distinguished from that in the bulk solution. This provides an opportunity to enhance water transport through the interaction between the molecules and the wall of the nano-channel walls. The ion exclusion capability of most 1D nanostructures is conferred by their permanent polarization and inner surface charge, which has in turn resulted in steric hindrance and electrostatic repulsion [61].

### 2.3. Two-Dimensional Nanomaterials

Two-dimensional (2D) nanomaterials, which have been known as the thinnest materials, are characterized by a layered structure configured as a honeycomb, governed by strong in-plane bonds and weak interlayer van der Waals forces [62]. The layered structure of 2D nanomaterial serves as the building block for the construction of nanofluidic channels that can be suitably applied for catalysis, biosensor and electrochemical energy-conversion processes. Mono- and few-layer nanosheets can be conveniently produced by physically or chemically exfoliating their van de Waals layered parent bulk counterpart. The typical example is the exfoliation of graphite to obtain graphene. Graphene is one of the most important and widely used 2D nanomaterials. After many successful implementations of graphene-enabled materials and devices, more 2D nanomaterials such as hexagonal boron nitride (hBN) nanosheet, layered double hydroxides (LDH), layered transition metal dichalcogenides, and Mxene have been engineered. 

Single-layer graphene is described as a nanosheet made from the planar arrangement of carbon atoms to form a hexagonal network. GO is described as an oxidized graphene sheet with basal planes decorated by functional groups such as carboxyl and epoxides, has been extensively used for separation processes [63]. The chemically bound functional groups expand the interlayer spacing to a desired distance, enabling selective and fast diffusion of small molecules. hBN, also known as white graphite, structurally resembles graphite hence share similar structural and physical properties with graphite [64]. Mxene is a relatively new class of 2D nanostructure composed of transition metal carbides nitrides and carbonitrides. MXene features the structure inherited from its parent MAX, which is a large family of hexagonal layered P63/mmc symmetry connected by strong metallic, ionic, and covalent bonds. Like GO, Mxene nanosheets are terminated with functional groups such as –H, –O and –F, allowing facile post-synthesis chemical functionalization [27]. Although Mxene and graphene share many common characteristics due to similarity in their structure, Mxene is known to show better compatibility with a polymer matrix, which is advantageous for nanocomposite membrane application [65]. LDHs are commonly known as hydrotalcite-like compounds composed of layered hydroxides of divalent (M^2+^) and partially substituted trivalent (M^3+^) cations with similar ionic radii. Compared to other 2D nanostructures, the multiple metal cations in LDHs allow precise control of chemical compositions of host layers [66]. Additionally, the substitutable charge compensating anions contribute to adjustable interlayer spacing and surface functionality. 

With their well-defined nanochannels and sub-nanometer pore structure, 2D materials offer precise and selective molecular separation capability. As one of the most popular choices for the development of nanocomposite membranes, the atomic-scale thickness of 2D nanostructures impart negligible transport resistance which renders fast permeation to realize high-throughput separation that is commercially attractive. Due to the structural advantage of 2D nanomaterials, pores can be directly created on the single layer nanosheets to form freestanding membranes with an ideal separation efficiency. The major technical challenge for obtaining such freestanding membranes is the difficulty of achieving uniform pore size distribution across the membrane area targeted for practical industrial applications. With more positive progress made in the synthesis and exfoliation method such as freeze-thaw exfoliation to obtain high-quality rigid crystals [67], crystalline 3D nanostructures with intrinsic nanopores have been constructed in 2D to resolve the issue related to uniform pore creation. One representative of this class of material is a 2D metal organic framework (MOF). There have been growing interest in the functionalization of 2D materials. The 2D nanochannels of sheet-like nanostructures can be easily decorated or intercalated with other functional materials to tune their physicochemical properties, impart additional functionalities or flexibility of the nanochannels to expand their application and achieve greater performances [68,69]. The interlayer composition enables structural control within the layers, where the ion or molecule selectivity and stability can be enhanced accordingly [70]. 

### 2.4. Three-Dimensional Nanomaterials

In contrast to the aforementioned low-dimensional 0D, 1D and 2D nanostructures, 3D nanostructures are not characterized by a well-defined geometry. A 3D structure can be simply constructed by either connecting several building blocks that extended in all directions or combining the 2D sheet-like structure to other building blocks [71]. On the other hand, crystalline nanoporous structured materials with a rigid cage, framework, or cavities can be generally classified as 3D nanomaterials. Other examples of 3D nanostructures include aerogel, foams, fibers, pillars and layered skeletons [16,72]. In most 3D porous structures, various building blocks can be rationally engaged and synergized. By tuning the 3D porous size and introducing other functional groups within the 3D compartments, the materials can be feasibly endowed with new multifunctional properties [73,74]. 

The microporous structures of these porous 3D nanomaterials provide channels for molecular transports. Known for their microporous crystal-like alumino-silicates frameworks in a 3D network, zeolites have been extensively used as nanofillers, particularly for gas separation nanocomposite membranes [75,76]. The 3D arrangement of SiO_4_ and AlO_4_ tetrahedra connected by oxygen bridges forms an open framework with pores and cavities. With the uniform pore structure and a controlled channel diameter, zeolites have been proven as excellent molecular sieves that able to perform selective sorption [77] and diffusion of ions and molecules [78,79]. As zeolite micropores possess diameters that are similar to the size of many molecules, they can precisely discriminate between molecules with just a small size variation. Lately, new zeolitic materials with enhanced pore accessibility and higher mesopore surface area have been explored. These include nanozeolite with their crystal size reduced below 100 nm and hierarchical zeolites introduced with secondary porosity in the mesopore range [80]. 

MOFs emerged as an alternative to zeolite and other conventional mesoporous materials. MOFs are crystalline porous structure with a hybrid array of metal ions or cluster coordinated to organic ligands [81]. Due to their porosity, uniform aperture distribution and tunable pore functionalities, MOFs have been widely used for molecule discrimination via gas adsorption and separation [82]. The connectivity and interactions of the cavities with target guest molecules can be controlled by altering the organic and inorganic building blocks [83]. The zeolitic imidazole framework (ZIF) is a sub-class of MOF which has been commonly used for nanocomposite membrane [84,85,86,87,88]. Although the MOF has been conventionally synthesized as a isotropic bulk crystal with a 3D network, nanosheet and nanofilm MOFs are gaining popularity owing to the merits stemming from the 3D porous framework and 2D intrinsic feature [89]. The covalent organic framework (COF) is a class of crystalline porous polymer that structurally resembles MOF, but it is made of light elements such as hydrogen, boron and carbon instead of metal-based nodes and organic components in the latter [90]. The porous network structure of COF which established through the periodically extended covalent bonds is characterized by high-density and well-arranged pores as well as uniform pore size [91]. COF can be formed in eclipsed and staggered 3D structures with tunable pores. Although MOFs have been exploited in more separation applications, with more efforts made in increasing the thermal and solvent stability of COFs [92], this new class of nanostructures has also been progressively used for nanocomposite membrane preparation [93,94]. Polyhedral oligomeric silsesquioxanes (POSS) belong to the group of silsesquioxanes with a general formula (RSiO_1.5_)n, where R is hydrogen or an organic group such as alkyl, aryl, alkylenes and their derivatives [95]. POSS has been commonly described as a 3D cage-shaped molecule composed of silica framework coordinated to multiple organic functional groups. Compared to other inorganic nanostructures, the organic linker functional groups in nanostructured hybrid inorganic-organic materials such as MOF and POSS have good affinity with polymers, hence facilitating better compatibility and interaction with polymer chains [96]. The polymer-nanofiller compatibility issues in the preparation of nanocomposite membranes can therefore be alleviated [97]. 

## 3. Tailoring the Dimensions of Nanostructures to the Separation Processes

The applications of various nanostructures have progressed from multiple aspects, including the fundamental understanding of water transport and molecule/ion-filler interaction through computational studies, separation performance evaluation through experimental approaches, modifications and functionalizations of nanofillers for performance enhancement. Due to their respective uniqueness and advantages originated from their structural properties and chemical composition, various nanomaterials of different dimensions have received equal attention in nanocomposite membranes. Just as the choice of the type polymer is largely dependent on the nature of the separation process, the selection of nanomaterials used for the preparation of nanocomposite membranes should also be made on the basis of their intended applications [98,99]. Tremendous efforts have been made in the preparation and performance evaluations of various forms of nanocomposite membranes. In this section, the discussion is made based on some recent exemplary work which aims at harnessing the benefits arisen from the uniqueness of the nanostructure dimensions, instead of the benefits rendered by their chemical compositions or surface properties. Out of the vast applications of membrane processes, two niche areas where this technology has been massively explored, i.e., gas separation and liquid separation are focused. 

### 3.1. Gas Separation

Dimensionally different nanostructures are introduced as a nanofiller, interlayer and coating layer of gas separation nanocomposite membranes [100]. Improved selectivity, permeability, durability and mechanical strength can be afforded by exploiting the structural advantages of many nanomaterials. In general, tubular and lamellar structures are used for facilitating the transport of gas molecules, while porous nanostructures with regular and well-defined openings or apertures are used to improve selectivity based on their molecular sieving properties [101]. 3D nanomaterials with rigid cage-like structure offer some structural advantages that are important for a gas-separation nanocomposite membrane. The last decade has witnessed tremendous efforts in developing MMM gas separation membranes based on 3D zeolite [102,103,104], MOF [105,106,107] and POSS [108,109,110]. In particular, MOFs exhibit a strong affinity towards CO_2_ molecules and are able to enhance CO_2_ solubility in the membrane hence offering excellent CO_2_ separation performance [111,112]. Great efforts have been devoted to reduce the particle size of MOFs and zeolite to improve their compatibility with the polymer matrix [113] and to introduce building units with specific gas adsorption capacity [114,115]. When incorporated into a polymer matrix with high free volume such as glassy polyacetylenes, cage-like POSS act as rigid barrier rendering a space-filling effect to reduce the relaxation of a polymeric chain and collapse of free volume cavities [116]. The large free volume occupied by a 3D POSS can effectively suppress physical aging. However, despite the availability of open cage structures, the large free volume is not accessible to the gas molecules [117]. As no concrete evidence has been found on the transport of penetrates across it, the transport behaviour of POSS is normally regarded as a 0D nanosphere. 

**Figure 3:**
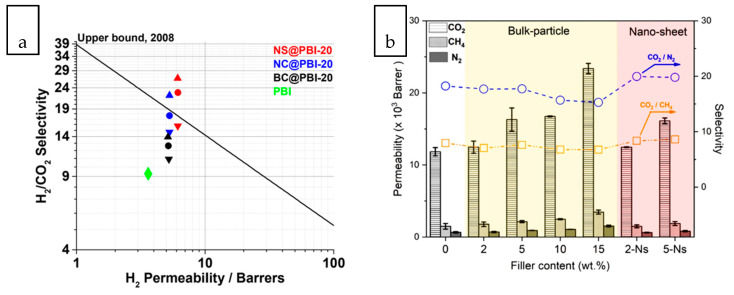

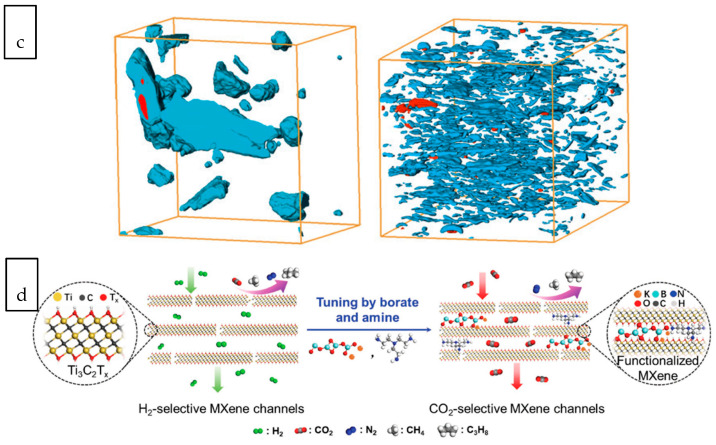
(**a**) H_2_ permeabilities versus H_2_/CO_2_ selectivities mixed matrix membranes (MMMs) containing 20 wt% of MOFs of different dimensions benchmarked with neat polybenzimidazoles (PBI) membrane (NS: nanosheet; NC: nanocube; BC: bulk crystal) [121], (**b**) CO_2_/CH_4_ separation performance of MMM incorporated with MUF-15 crystal bulk and nanosheets [122], (**c**) Segmented (focused ion beam- scanning electrone microscope (FIB–SEM) tomograms of polyimide-based MMM containing bulk-type and nanosheet CuBDC MOF [123], (**d**) Schematic illustration of the interlayer functionalization of MXene to tune from a H_2_-selective to CO_2_-selective membrane [124].

Realizing the intrinsic benefit of anisotropic material, there has been increasing interest over the last few years in using lamellar zeolite and MOF as the nanofiller of nanocomposite membranes [118,119,120]. These studies confirmed the role of 2D lamellar morphology to render a perpendicular pore orientation which can shorten diffusion paths for the desired components but increase the tortuosity of the undesired components. Kang et al. correlated the H_2_/CO_2_ separation performance of MMM with the dimensional properties of a small pore MOF nanofiller, namely [Cu_2_(1,4-naphthalene dicarboxylate)_2_(1,4-diazabicyclo[2.2.2]octane)]_n_ in the form of bulk crystal, nanocube, and nanosheet [121]. The nanosheet MOF exhibited the highest CO_2_ uptake owing to narrow MOF channels which has provided homogeneous sorption sites for CO_2_. Compared to bulk crystal MOF, nanocube MOF distributed more uniformly throughout the matrix due to the reduced particle size. Nevertheless, MMM containing nanosheet MOF has exhibited the highest H_2_ permeability and H_2_/CO_2_ selectivity, as depicted in Figure 3a. The main reason lies in the partial stacking of MOF nanosheets, due to the shear force imparted during membrane casting, which serve as CO_2_ barriers to enhance the sieving efficiency. A relatively new type of MOF, MUF-15 in bulk crystal and nanosheet geometries was introduced into a PIM-1 polymer matrix for CO_2_ separation [122]. The MUF-15 nanosheets offered large interfacial contact area between the polymer and filler phases to enhance compatibility while interfacial voids were formed when MUF-15 bulk crystal was used. As a consequence, CO_2_/CH_4_ selectivity enhancement was achieved with the former nanofiller, as depicted in Figure 3b.

Compared to the 3D analogue with bulkier network cage, 2D MOF can be favorable used for film formation and surface coating [125]. When used as the dispersed phase of a nanocomposite, the 2D geometry provides larger contact surface and improves the adhesion between the MOF and substrate or host matrix. It has been observed that the MOF nanosheet could disperse uniformly throughout the polymer matrix as sheet-like structures have larger contact area with polymer matrix compared to isotropic morphology. The FIB–SEM tomograms shown in Figure 3c compared the distribution of bulk crystal MOF and nanosheet MOF of same loading within the polyimide matrix [123]. A striking difference was observed where the bulk crystal left a significant fraction of unoccupied volume accompanied with non-selective voids while lamellar MOF occupied a large fraction of the volume in very uniform pattern. The surface area of nanosheets embedded in the polymer matrix was nearly 10 times greater than that of the bulk crystal counterpart of same loading of filler, which improved molecule sieving efficiency by up to 80%.

Facilitated transport membrane is important for gas separation as it enables excellent permeability and selectivity for a targeted gas through the incorporation of a carrier agent with a special affinity toward a target gas component. In this context, the 2D nanosheet and 3D nanoporous crystals are beneficial as the interlayer spacing between nanosheets and porous framework structures can readily host the carrier agents. An interesting chemical functionalization has been demonstrated by Shen et al. to tune the gas selectivity of MXene nanofilm horizontally aligned on a substrate [124]. As illustrated in Figure 3d, the interlocking of borate and polyethylenimine within the nanoconfined MXene nanosheets reduced the interlayer spacing. The chemical functionalization of CO_2_-philic borate which act as CO_2_ facilitated transport carrier transformed the nanoconfined layers of Mxene from diffusion-controlled to solution-controlled channels. As a result, the membrane was tuned from a H_2_-selective to CO_2_-selective membrane. Water plays an important role in CO_2_ facilitated transport. When incorporated into the polymer matrix, 2D nanosheets with large surface area-to-volume ratio contributes vast space to redistribute the water domain present in the humidified membrane such that water-rich domains are available throughout the matrix to facilitate the sorption of CO_2_ [126]. The nanosheets can also effectively disrupt and induce reorientation of polymer chain packing which in turn increased gas diffusion.

In terms of the current development of polymeric membranes for gas separation, equal attention has been dedicated to both integrally skinned asymmetric membranes and thin film composite (TFC). However, for practical industrial application, TFC membranes can offer several advantages over the integrally skinned asymmetric counterpart [127]: (i) the construction of thin selective layer increases the gas permeation and overall productivity, (ii) only small amount of material is needed for the construction of the selective layer, resulting in material cost saving, (iii) independent optimization of each layer in the composite membrane. When a nanocomposite membrane is put into this context, TFN offers another significant advantage where the nanomaterials can be concentrated at the selective layer, instead of throughout the polymer matrix such as in the case of MMM, to minimize the wastage and maximize the functionalities of the selected nanomaterials [128]. Bearing in mind these structural benefits, TFN membranes are expected to be more commercial-ready compared to the MMM counterpart. As forming an intact selective layer is a top priority during the preparation of thin film membranes, the interaction of the incorporated nanomaterials and the polymer host matrix is of great importance. Bearing in mind this concern, it is not surprising that 2D nanostructures appear as the most favourable nanofiller for gas separation TFN membranes. Compared to 1D nanostructures with high aspect ratio and 3D nanostructures with rigid interconnected framework structure, 2D nanosheets can be more flexibly aligned to form highly compact layered structure or used as interlayer in the construction of TFC [129].

### 3.2. Liquid Separation

**Figure 4:**
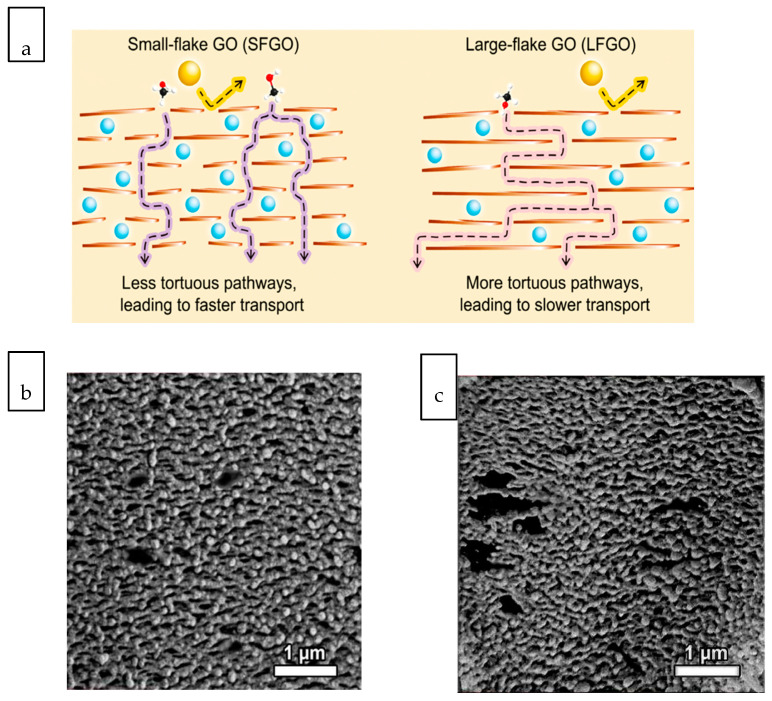
(**a**) Schematic illustration of solvent molecule transport pathway in small-flake graphene oxide (GO) and large-flake GO with different lateral [133], cross-sectional images of polyethersulfone (PES) support layers templated with (**b**) ZnO nanoparticles and (**c**) ZnO nanorods [134].

Ultrafast water transport is the ultimate goal of all liquid separation processes. Increasing membrane water permeability can potentially decrease the membrane surface area requirement and the associated costs [130]. The water permeability of membrane has also been associated with the specific energy consumption of a pressure-driven membrane process such as reverse osmosis [131]. As most commercial membrane processes have demonstrated satisfactory separation efficiencies, the application of nanocomposite membranes for liquid separation is largely related to their potential in enhancing water permeability while retaining the selectivity, primarily due to the structural features of inorganic nanomaterials. On account of their tubular or nanoporous layered structures, 1D and 2D materials are especially attractive to facilitate high water permeability across the nanocomposite membranes. As the separation mechanism of 2D nanosheets is primarily governed by the distance or tortuosity of the transport pathways, the control of lateral dimension of 2D nanofillers is useful to maximize the transport capacity [132]. By manipulating the exfoliation conditions, small- and large-flake GO nanosheets with lateral dimension of 0.03 μm^2^ and 0.51 μm^2^ can be obtained [133]. Using methanol as a model fluid, the nanocomposite membrane assembled with small-flake GO exhibited up to 2.7-fold higher permeance than the large-flake counterpart. The small lateral dimension of small-flake GO enabled a less tortuous pathway which led to faster transport, as illustrated in Figure 4a. However, despite the advantage of the small-flake nanosheet, forming a homogeneous thin film of small-flake GO on top of a polymeric substrate was found to be challenging as the larger substrate pore size could not effectively retain the laminate in a localized region.

Nanomaterials with various dimensions have been used as pore forming templates to tailor the nanostructure of polymer substrate [135,136]. In this direct template technique, etching agents are used to remove the nanomaterials preloaded into the nanocomposite membranes, leaving templated porous structure with increased porosity and pore space connectivity. Lu et al. compared the templated porous structure of forward osmosis (FO) polysulfone (PSf) substrate formed upon the washing of 0D LDH spherule and 3D LDH flower from the nanocomposite membrane [137]. Similarly, Rastgar et al. introduced 0D ZnO nanoparticle and 2D ZnO nanorods within PES FO substrate to create secondary pores as shown in Figure 4b upon the washing-off of these nanostructures [134]. 0D spherical nanotemplate with particle size <100 nm cannot effectively interconnect the isolated pore space. The substrate templating using 3D LDH and 2D ZnO nanorod could more effectively increase the overall porosity and interconnected pore network, which in turn favoured the water diffusion through the specific channels and suppressed internal concentration polarization (ICP) in the FO membrane.

Particle size and surface area play important roles in the interaction of materials with its surrounding medium or substances [138]. These feature are especially relevant to two relatively new classes of functional nanocomposite membranes used for wastewater treatment, i.e., photocatalytic membrane and adsorptive membranes [139]. For both photocatalytic membranes and adsorptive membranes, as the photocatalyst and nanoadsorbents, respectively, cannot be wholly exposed to the contacting medium like when they are suspended in the medium, the effective surface area available for the reaction or interaction is greatly reduced. In order to maximize the efficiency of the photocatalysts and nanoadsorbents in the nanocomposite structure, it is a priority to optimize the important features of the nanomaterials prior to their incorporation into the polymeric matrix. The effect of adsorbent dimension on its specificity toward certain types of metal ion has been evidenced. By comparing the adsorption behavior of 0D and 2D silica-based nanoadsorbents, Diab et al. observed that the 2D montomorillonite exhibited higher uptake efficiency of Zn^2+^ while 0D silica nanoparticle was more specific towards Cr^3+^ [140]. The discrepancy was attributed to the interaction of sheet-like and spherical like nanostructures with the metal ions of different ionic radii. For nanomaterials featuring antimicrobial properties, their applications in nanocomposite membrane are interesting to mitigate biofouling. Myriads of nanomaterials including AgNP and single-wall CNTs, have been incorporated into polymer matrix to afford antimicrobial properties [141,142]. Besides the chemical nature of the constituents, the intrinsic biocidal potency of the nanomaterials is also closely related to their dimensions which arise from a variety of particle sizes, shapes and aspect ratios. A comparison of the antibacterial activity of ZnO nanosphere and nanosheet over *E. coli* and *S. aureus* bacterial species evidenced that nanosphere demonstrated higher antimicrobial efficiency due to the better penetration into the cell wall of bacteria [143]. On the other hand, it has also been observed that despite having the same chemical composite, sheet-like RuO_2_ showed more inhibitive capability towards Gram-positive bacteria compared to its spherical counterpart [144]. The discrepancy between these studies can be attributed to the difference in their antibacterial mechanisms [145].

## 4. Synergy of Multidimensional Hybrid Nanostructures

To further enhance the performance, more than one type of nanostructure has been simultaneously used for the construction of the membrane structure. When nanostructures with different dimensions are used, the difference in the geometric structures allows for their distinct roles in the nanocomposite membrane design. The integration of two or more nanostructures with different geometrical structures can also potentially diminish the limitation of individual component. For instances, the decoration of 1D nanoparticles on 2D materials such as graphene and MXene, which serve as the backbone, alleviate the agglomeration issues [146,147]. The interlayer of 2D materials can also be integrated with 1D nanomaterials which act as spacer to reduce the restacking tendency of nanosheets. There are two ways that two or more nanomaterials can be introduced into the nanocomposite membranes, i.e., hybridization of two or more nanomaterials as a new single entity prior to their integration with the membrane or simultaneous introduction of two or more individual nanomaterials during the membrane preparation. 

**Figure 5:**
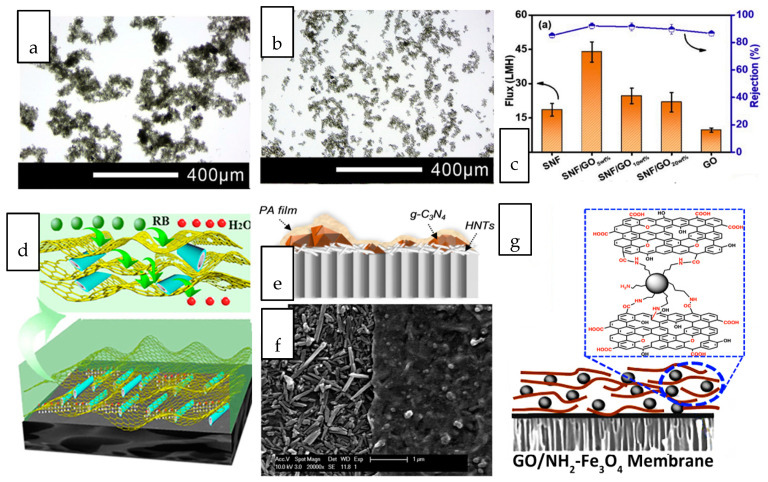
Optical images of (**a**) carbon nanotube (CNT) and (**b**) CNT/GO dual nanofiller dispersed in aqueous-based solution. (**c**) nanocomposite membranes incorporated with SNF/GO_x_ exhibited drastically improved Na_2_SO_4_ rejection and flux for the SNF, GO, and membranes [156] (**d**) conceptual illustration of water transport path and nanocapillary network jointly created by porous reduced graphene oxide/halloysite nanotubes (PRGO/HNTs) (RB: Reactive Black) [157] (**e**) Schematic illustration of cross section and (**f**) surface image of TFN membrane incorporated with g-C_3_N_4_/HNT. The left part with the PA scraped off revealed the orientation of HNT [158], (**g**) Schematic illustration of NH_2_-Fe_2_O_3_ intercalated GO nanosheets [159].

The incorporation of dual nanostructure or hybrid composed of two of more dimensionally different nanostructures into gas separation membranes have been ventured by numerous studies [148,149,150,151,152,153]. Wong et al. investigated the synergistic effects of nanotubular and nanosheet structures on the formation of an interfacially polymerized polyamide layer [154]. When the dual-fillers were dispersed in their common solvent, GO nanosheets with high dispersibility served as a dispersant for the CNTs in aqueous solution hence preventing the latter from aggregating (Figure 5a,b). The deposition of nanotubes onto the basal plane of GO reduced the tendency of nanotube agglomeration. The combination of these 1D and 2D nanostructures led to the formation of a thicker and rougher polyamide layer compared to that of incorporated with single-filler. In terms of the gas separation performance, the nanocomposite membranes harnessed the high selectivity offered by CNT and the high permeability contributed by GO, hence exhibiting the optimal CO_2_ permeability, CO_2_/N_2_ and CO_2_/CH_4_ selectivity that which are 29.7%, 63.5% and 54.1% higher than that of pristine TFC membranes. By coupling 2D MXene and GO with silica microsphere and HNT, Shi et al. demonstrated the importance of identifying the right pair of nanofillers to achieve the desired synergistic dual-filler effects [155]. The Pebax-based nanocomposite membrane incorporated with GO/HNTs dual-fillers exhibited much higher CO_2_/N_2_ selectivity than the nanocomposite membrane embedded with MXene/HNTs at the same loading. The findings stem from the different rigidity of GO and MXene where HNTs were expected to be better wrapped by the flexible GO sheets to promote their dispersion. The preferential horizontal orientation of GO and HNTs improved the tortuosity of gas transport and hence increased the diffusivity selectivity of CO_2_/N_2_. In contrast to GO, MXene/SiO_2_ dual fillers demonstrated synergic effect that could not be observed in MXene/HNT. The dispersion of spherical SiO_2_ was much better than that of HNT, hence can effectively prevent the stacking of MXene.

Various interesting combinations of nanostructures with different dimensions, including the coupling of 0D/2D nanostructures [160,161,162,163,164,165,166,167,168,169], 1D/3D nanostructures [170], 1D/2D nanostructure [171,172,173], 3D/2D nanostructures [174,175,176,177,178] have been attempted for liquid separation nanocomposite membranes. A glimpse into these works revealed the huge potential of high surface area 2D nanosheets to serve as a versatile platform for the deposition of other nanostructures while their restacking issue can be simultaneously overcome through the insertion of foreign nanostructures. The synergistic advantages of 1D silk nanofiber (SNF) and 2D GO in the nanocomposite membranes over their single counterparts when used for salt and dye separation has been demonstrated [156]. Supported on a hydrolyzed polyacrylonitrile support, the silk nanofibers intercalated between GO layers, forming organic-inorganic stackings. The flux and salt rejections of the nanocomposite membranes incorporated with SNF/GO were increased by up to 80% compared to those of incorporated with individual SNF and GO (Figure 5c). The 1D silk nanofiber interspersed between the 2D GO layers inhibited the extension of GO nanosheets thus maintaining the salt rejection capability. The additional space created by the SNF interspersed between the GO nanosheet layers led to an optimal increase in the membrane free volume, creating more flow path for water transport. Sheet-like porous reduced graphene oxide (PRGO) and tubular halloysite nanotubes (HNT) were used to create a continuous sandwich-like water channel for efficient dye removal [157]. The driving force induced during solvent-evaporation coating facilitated the movement of HNT to the interlayer spacing of two adjacent PRGO sheets, forming a network of nanocapillaries as illustrated in Figure 5d. 2D graphitic carbon nitride (g-C_3_N_4_) and 1D HNTs were co-deposited onto a substrate through vacuum filtration prior to interfacial polymerization of polyamide selective layer [158]. Due to the distinct dimension of these nanostructures, the tubular HNTs inclined to horizontally oriented on the substrate surface to act as an interlayer, while g-C_3_N_4_ particles scattered within the PA layer as a porous nanofiller, as revealed in the surface morphology shown in Figure 5e,f. The intrinsic nanopores across the lamellar g-C_3_N_4_ and nanotubular structure of HNTs provided additional water transport pathways, which eventually led to elevated water permeability up to 20.5 L·m^−2^·h^−1^·bar^−1^ while maintaining the salt rejection capability. 

Zero-dimensional nanoparticles are favourably used as intercalating agent for sheet-like nanostructures due to their good dispersibility throughout the interlayer spacing. Amine functionalized Fe_3_O_4_ nanospheres were intercalated within the interlayer of GO (Figure 5g) to enlarge the interlayer spacing and improve the water stability of the GO-coated membranes [159]. The nanocomposite membranes augmented by Fe_2_O_3_/GO exhibited a water flux of up to 78 L·m^−2^·h^−1^ in dye/salt separation, which was nearly 5 times greater than that with single GO. Three-dimensional nanostructures have been increasingly used for intercalation of nanosheets. The precisely controlled framework and cage-like structures of many 3D nanomaterials can offer additional sieving capabilities without hindering the passage of water molecules. Unlike impermeable nanostructures, the opening of nanoporous crystals with 3D channels can increase the porosity and introduce more nanofluidic channels [179]. By incorporating UiO-66 nanoporous crystals with sub-nano aperture size into the reduced graphene oxide (rGO) laminates, the result exhibited 15 times higher water permeability than the nanocomposite membrane incorporated with rGO without compromising the dye removal efficiency [180]. The improvement was attributed to the abundant adsorption sites and extra water paths rendered by the 3D/2D hybrid. 

## 5. Anisotropy and Orientations of Nanostructures

Nanostructured materials with high aspect ratios or extended lateral dimensions are endowed with exciting physicochemical properties that are radically different from those isotropic counterparts. However, these unique direction-dependent properties can only be harnessed if the orientation of these anisotropic nanostructures is taken into consideration. The preferable orientation of the nanomaterials not only may exhibit properties superior to the disordered counterparts, but also maximize the anisotropic properties of the nanomaterials to approach their ideal performance [181,182]. As the alignment of 1D and 2D nanostructures in their preferable direction is important to fit their purpose, the directional alignment of anisotropic nanomaterials in the nanocomposite membranes should be given particular attention. In the majority of cases, the incorporated nanomaterials are assumed to be randomly orientated and uniformly dispersed within the polymer matrix. The enhancement in separation properties are generally discussed based on the overall physicochemical properties contributed by the nanomaterials, rather than the advantages arisen from the structure or dimension of the selected materials. 

**Figure 6:**
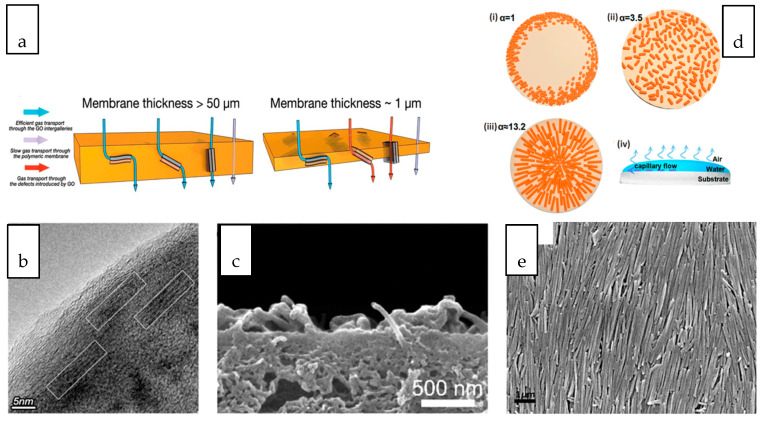
(**a**) Schematic illustration of gas transport pathway across membrane with different thickness [184] (**b**) Cross-sectional image of GO nanosheets aligned at parallel direction with membrane surface [184] (**c**) Cross-sectional images of nanocomposite membranes showing the protrusion of the tubes [185]. (**d**) Schematic illustration of nanomaterials with different aspect ratio (α) (i) coffee-ring effect observed in isotropic nanostructures, (ii) uniform distribution of ellipsoid nanostructures (iii) orientation of nanotubular nanostructure, (iv) evaporation deposition of nanomaterials on a substrate [186]. (**e**) Surface image of oriented HNT [186].

Tubular and sheet-like nanomaterials, when oriented in different directions within the polymer matrix, result in different gas transport behaviors. As depicted in Figure 6a, for composite membranes with a relatively thick selective layer, GO nanosheets aligned perpendicular to the membrane surface facilitated the diffusion of gas molecules through the interlayer spacing of nanosheets while that aligned parallel to the membrane surface lengthens the pathway for the passage of gas molecules although the gas molecules still preferably transported through the GO interlayer spacing [183]. However, despite this ideal orientation, the practicability of the membrane should also be taken into consideration. Formation of thin (~1 µm) selectively layer that is preferable for a practical separation process may not favour the vertically or randomly oriented GO nanosheets considering the defects and voids created. Zhang et al. aligned GO horizontally in the thin PEBAX selective layer (Figure 6b) by taking the advantage of shear effects during dip-coating process [184]. Although the tortuosity of the gas transport pathway has been increased upon the addition of GO, the parallel-aligned GO laminates provide size-selective and fast gas transport channels without creating defects within the selective layer. Compared to the randomly oriented and neat polymeric membrane, the nanocomposite membrane with parallel-aligned GO demonstrated improved CO_2_ permeance without compromising the CO_2_/N_2_ selectivity.

When 1D CNT emerged as one of the most studied nanofillers for nanocomposite membranes, the astonishing transport behaviour modelled from vertically aligned CNT has evoked several breakthrough efforts in preparing nanocomposite membranes with vertically-aligned CNT. The vertical alignment has been realized through an infiltration technique which involves the wetting and infiltration of the vertically aligned CNT array by the chosen polymer solution to fill up the inter-tube gaps [187]. The approach may not be favourable for large-scale preparation but it has stimulated more interest in exploring other scalable technique to accomplish the alignment of CNT within nanocomposite membranes. The magnetic and electrical responsiveness of CNT makes the application of a unidirectionally magnetic and electrical field a promising technique for facile nanotube alignment [188]. Polyamide NF TFC membrane with electrically-aligned CNTs that were vertically spanning from polysulfone substrate to polyamide selective layer demonstrated a several-fold increase in the water flux [185]. However, by considering the thickness of membrane and the length of CNT which normally ranges from a few microns to a few centimeters, the length of vertically aligned CNT must be carefully controlled to avoid protrusion of the tubes, as shown in Figure 6c [185]. In another study, the orientation of aluminogermanate imogolite nanotube to the direction of the water flow path within the polyamide layer of TFC membrane was evidenced as the primary factor contributing to the increased specific water flux [189]. The improvement was attributed to the reduction in the length of the water passage across the thin film. 

Solvent evaporation is a common technique employed for the formation of a thin film embedded with nanofillers [190]. The deposition and distribution of nanostructures during evaporation is a dimension-dependent process. The coffee-ring effect refers to the deposition of the suspended particulate in a ring-like fashion, mainly observed for the deposition of isotropic materials, such as the nanosphere [191]. Figure 6d compares the deposition patterns of nanostructures of different aspect ratios [186]. It is observed that the deposition of nanostructures with anisotropic geometry through evaporation can significantly deform the interfaces and produce strong interparticle capillary interactions. These deformations are responsible for the elimination of the coffee-ring effect where uniform deposition of the nanostructures can be achieved. The uniform distribution and alignment is more significant with the increasing aspect ratio of the nanostructure, as evidenced in the orientation of HNT on the polyacrylonitrile substrate through evaluation (Figure 6e) [186].

## 6. Gaps and Perspectives

Developing a high-performance membrane would largely heighten the separation performances and sustainability of a membrane process. The nanocomposite membrane is currently at the forefront of membrane research and development, owing to its unparalleled physicochemical properties capable of resolving the underlying issues of conventional membranes. It is without any doubt that nanomaterials offer a bridge between atomic characteristics and macroscopic devices or materials. In parallel with the advancement in nanocomposite membranes, there is a need to look into the architecture and design of the material. For nanocomposite membrane development, the focus has been largely placed on exploring various fascinating nanomaterials to break the boundaries between laboratory attempts and commercial application. The main challenge of current material selection for the development of a high-performance nanocomposite membranes is that there are too many potential candidates available. Dozens of new materials of various geometrical structures and their allotropes have emerged in the past few years. With many more anticipated to exist, the selection of material obviously cannot be fulfilled by simply trying thousands of existing materials. A strong integration of theories and the understanding of the characteristics of materials and their resulting properties is crucial to promote good matching and design of new materials that suit their applications well. A true understanding the material properties is a gateway for achieving more significant breakthroughs in material developments. Additionally, the membrane development cycle can be effectively shortened while the benefits can be fully optimized. 

Albeit the fact that great progress has been made in exploring the potential of new nanomaterials to enhance the performance of membranes for liquid and gas separations, there is still plenty of room for improvement. Along with the emergence of more exciting nanomaterials that can be potentially used for nanocomposite membranes, it is worth putting more effort into understanding and refining the properties of the existing materials. There are three common ways to tune the properties of nanostructures, i.e., by altering the chemical composition, surface functionalization and structure/morphology. These alterations not only lead to the corresponding changes in their physicochemical properties, but also functionalities that are responsible for their applications. While the research topics related to surface functionalization have been an evergreen favourite, the importance of understanding the role of dimension of the nanostructures in designing high-performance nanocomposite membranes should not be overlooked. 

The versatility of altering the dimension of a performance-proof nanostructure to furnish other desired properties is an interesting strength to intensify the applicability of these nanostructures for the preparation of nanocomposite membranes. By taking carbon-based nanomaterials as an example, the majority of the carbon allotropes are engineered and artificially synthesized in the laboratory. Therefore, the nanostructures enjoy high versatility for further modification, in both in situ or post-synthesis stages. This flexibility stimulates more innovation in nanomaterial synthesis and modifications. The better control and understanding in a top-down exfoliation strategy or bottom-up assembly methods allow the alteration of nanostructure dimension, from what it is commonly known to be. One vivid example is the conversion of a 3D rigid MOF to 2D flexible network to address the limitations of the former [89]. The diffusivity and accessibility of molecules to the active sites of the MOF can be improved without compromising the chemical functionality as well as chemical stability demonstrated by the 3D analogue. Such a fascinating 2D material has been recently explored for nanocomposite membranes where their superior properties arising from the 2D structure are evidenced from the improved performance. Interestingly, 2D graphene-based nanostructures have been architectured into 3D macrostructures such as in the form of graphene hydrogel and graphene sponge [192]. A three-dimensional microporous graphene network has been topologically synthesized with interconnected channels of zeolite as a template on account of their high porosity, large specific surface area and light weight [193]. The structural advantages have been witnessed in some applications, including for oil/water separation and gas separation. Although these materials are still not common for the development of nanocomposite membranes, the anisotropic properties of graphene-based materials deserve further attentions. Metal oxide nanoparticles which have been conventionally synthesized as 0D spherical nanostructures have also been increasingly explored in their 2D structures [26].

It is important to unlock the full potential of nanomaterials by matching their unique structural characteristics with the applications of the resultant nanocomposite membranes. For instance, the orientation of anisotropic nanofillers should not be neglected if they are intended for rendering mechanical reinforcement. Similarly, for many nanostructures that are targeted for facilitating molecular transport, more studies should be directed to more in depth understanding of the transport pathways within these materials. With more examples demonstrating the importance of directional alignment of anisotropic nanomaterials in affecting molecular transport pathways, more attention should be placed on looking into the possible ways to maximize the benefits of these nanomaterials. This review does not intend to delve in the alignment techniques but rather to pinpoint the necessity of putting more emphasis on understanding the effects of nanomaterial orientations on the behavior of the nanomaterials. This is especially important for 1D and 2D nanostructures that are intended for fast molecular transport. It is undeniably challenging to precisely tune the orientation of these nanostructured particularly when processed over a large membrane area. The molecular transport across a nanocomposite membrane is very different from that of polymeric or inorganic membranes as it integrates the motion of fluid through and around the nanostructure as well as across the polymeric or inorganic structure. In many experimental studies, the effects of nanofiller orientation on the molecule flow path across the nanocomposite membranes have been neglected. Although this topic remains a great challenge, closing the gaps will be a key step towards performance optimization. 

The hybrid of multidimensional nanostructures is an interesting route to magnify the structural advantages of the nanostructures and to mitigate the issues related to their processing such as dispersion and compatibility. Functional nanostructures with enhanced properties can be synergistically achieved from two or multiple components in the resultant hybrid. The synergistic effects revealed the infinite possibilities to fine tune the properties and functions of nanomaterials through judicious pairing of nanostructures. Compared to the confined tubular structure of 1D and spherical hollowless 0D structures, 2D nanosheets create a more versatile platform for the insertion of foreign materials between the two adjacent nanosheets. The current work majorly focuses on the binary hybrid of nanostructures of the same dimension or different dimensions. Ternary or quaternary hybrid nanostructures with properties that are expected to surpass that of the binary should be given more attention in future research. While synergistic effects are generally observed in most dual-filler or hybrid systems, both experimental and molecular simulation studies are needed to provide further clarifications and insights regarding the role of each component. By taking the MOF/GO hybrid as an example, although it has been agreed that the water flux enhancement was mainly attributed to the increase in the GO interlayer spacing as a result of MOF intercalation, a recent computational study has pointed out that the high water permeability can only be effectively achieved with high loading of MOF [194]. Such finding highlights the potential of exploiting the selective adsorption capacity, rather than the water channel, of MOF to augment the membrane performances. It is also rational to point out that, although enhanced separation performances have been reported with the co-existence of two or more fillers in the nanocomposite membranes, most descriptions are made based on the conceptual interactions between the hybridized components. Direct evidence of their interactions such as the arrangement of tubular nanomaterials between the interlayer spacing of sheet-like nanostructures has not been fully found with the existing characterization tools. 

Confusion arises when attempts are made to distinguish the roles of nanomaterials proffered by their geometries, physical properties and chemical compositions. While it is unnecessary to isolate the multiple factors that influence the nanomaterial properties, based on the exemplary findings discussed in this review, the dimension of these nanostructures has a disregarded role in determining the functions of the nanomaterials. Admittedly, for some nanomaterials, the unique physical properties induced from the dimensional advantages have not been truly exploited in the preparation of nanocomposite membranes. For instance, the quantum size effects associated to 0D nanostructures has marginal significance in the enhancement of nanocomposite membranes. However, the surface properties related to their chemical composition such as surface charges of metal oxide nanoparticles, antibacterial properties of AgNP and surface functionalities of GO quantum dots play more important roles in tailoring the properties of the resultant nanocomposite membranes. It would be a meaningful line of research if further exploration can be made to further verify the dimension-dependent properties of the nanomaterials. A direct comparison of the structural properties and separation performance of nanocomposite membranes prepared with the same material composition and experimental condition, but of different dimensions, will provide a clear indication of the roles of material dimensions. For examples, the characterization and performance evaluation of a series of photocatalytic membranes prepared with 0D titania nanoparticle, 1D titania nanosheet, 2D titania nanotube and 3D titania nanoshell would provide useful information regarding the interactions of these multidimensional nanostructures with the polymer matrix, the trend in textural, morphological and optical properties as well as the correlations of these factors with the photocatalytic and filtration performances of the resultant nanocomposite membranes. Such comparison not only gives a unified view concerning the effects of dimension, but will also be helpful for screening and selecting the optimal nanostructure at the preliminary stage. 

## 7. Conclusions

In this review, we have discussed the roles of nanostructure dimensions, specifically those based on their applications in nanocomposite membranes dedicated to gas separation and water reclamation. Despite the undeniable benefits offered by nanomaterials in the resultant nanocomposite membrane, there are many fundamental aspects which can be looked into. One of these is the role of the material dimensions. The classifications of nanomaterials based on their dimension serves as a useful guideline to screen a wide range of nanomaterials based on their structural properties. Nevertheless, proper inspection of their unique dimensional features is of utmost importance to avoid the mismatch between the nanomaterials and the purpose they serve when incorporated into the nanocomposite membranes. The discussions based on the exemplary works in gas and liquid nanocomposite membranes provided further insights into this aspect. The research gaps and perspectives on these lines have also been identified, some of which are a great challenge and remain to be further explored. The insights reached in the article can be extended beyond the materials that have been explored thus far. Nanocomposite membranes used for other purposes, for instance fuel cell and biomedical applications, will also become relevant if the roles of nanofillers are clearly defined. With many successful attempts acting as a driving force, it can be envisaged that the research and development of nanocomposite membranes in the field of separation will continue to expand in the coming decade.
